# Effectiveness of Virtual Reality–Based Simulation Training as a Supplement to Traditional Simulation Training for Improving Neonatal Resuscitation Performance Among Doctors and Nurses in Denmark: Protocol for a Multicenter Randomized Controlled Trial

**DOI:** 10.2196/93439

**Published:** 2026-07-24

**Authors:** Hanna Rahimi, Anja Poulsen, Jette Led Sørensen, Ida Madeline Hoffmann, Amalie Middelboe Sohlin, Line Klingen Gjærde, Stine Lund

**Affiliations:** 1 Department of Paediatrics and Adolescent Medicine, Copenhagen University Hospital North Zealand, Dyrhavevej 29, Hillerød, Capital Region, 3400, Denmark, +45 52441529; 2 Department of Paediatrics and Adolescent Medicine, Rigshospitalet, Copenhagen, Capital Region, Denmark; 3 Mary Elizabeth’s Hospital and Juliane Marie Centre, Rigshospitalet, Copenhagen, Capital Region, Denmark; 4 Department of Clinical Medicine, University of Copenhagen, Copenhagen, Capital Region, Denmark

**Keywords:** virtual reality, immersive technology, neonatal resuscitation, neonatal life support, simulation training

## Abstract

**Background:**

High-quality neonatal resuscitation (NR) depends on timely execution of technical and nontechnical skills. Simulation-based training improves NR performance, but it is resource-intensive and difficult to deliver at sufficient frequency. Immersive virtual reality (VR) simulation may provide a scalable supplement to traditional mannequin-based training; however, evidence from European neonatal training settings is limited.

**Objective:**

The aim of this study is to evaluate whether immersive VR-based simulation training used as a supplement to traditional mannequin-based simulation training improves NR performance among doctors and nurses in Denmark.

**Methods:**

NEONATAL is a multicenter, individually randomized, 2-arm controlled superiority trial with a parallel-group pretest-posttest design (trial registration number ISRCTN 43822066). Resident doctors and neonatal nurses from 4 hospitals in Eastern Denmark will be randomized to either traditional mannequin-based NR training alone or traditional mannequin-based simulation training supplemented with immersive VR simulation training. Outcomes will be assessed at baseline and endline at 6 to 8 weeks using standardized neonatal simulation scenarios, validated assessment tools, and questionnaires.

**Results:**

The study was funded in July and October 2025, as well as in January 2026. Participant recruitment began in October 2025 and was completed in December 2025, with 66 participants enrolled. Data collection was completed in February 2026. Data cleaning and analysis will take place from March to July 2026, and the results are expected to be published in autumn 2026. This study will provide data on the effectiveness, feasibility, and usability of immersive VR simulation training as a supplement to traditional mannequin-based NR training in routine clinical education.

**Conclusions:**

The NEONATAL study addresses an important gap in NR education by evaluating immersive VR simulation training as a supplement to traditional mannequin-based training.

## Introduction

Neonatal health is a crucial component of global child health. Despite advances in global child health, neonatal mortality remains a major challenge, accounting for nearly 47% of the 4.9 million under-5 deaths recorded globally in 2022 [1,2]. Preventable neonatal morbidity and mortality persist even in high-resource settings, particularly during the first minutes of life, when rapid and coordinated interventions by well-trained health care workers (HCWs) are essential. Up to 85% of newborns breathe spontaneously at birth, approximately 10% of newborns require basic interventions such as drying, warming, stimulation, or airway maneuvers, and around 5% require positive pressure ventilation (PPV) to support breathing. Fewer than 0.3% of newborns need advanced resuscitation, including chest compressions or medication [3].

Global initiatives, including the United Nations’ Sustainable Development Goals, aim to reduce under-5 mortality to at least 25 per 1000 live births and neonatal mortality to 12 per 1000 live births by 2030 [4]. The leading causes of neonatal mortality globally are prematurity, intrapartum-related complications (including birth asphyxia), and neonatal infections, all of which are preventable or treatable with timely, evidence-based care [5,6]. Achieving these outcomes requires HCWs who are not only knowledgeable but also able to translate skills into rapid, coordinated actions during emergencies, which remain a significant challenge [7-10]. In neonatal resuscitation (NR), patient outcomes are highly dependent on the HCWs’ ability to perform time-critical interventions, particularly airway management and initiation of effective PPV [3]. Timely initiation of ventilation is critical, as delaying ventilation by just 30 seconds increases the risk of adverse outcomes, including morbidity and mortality, by 16% [11]. Simulation-based training (eg, using mannequins) has demonstrated effectiveness in improving NR skills, team communication, and outcomes [12,13]. However, traditional training requires physical presence, trained instructors, and structured environments—which limits scalability in busy health care settings. As a result, maintaining proficiency remains challenging because NR events occur relatively infrequently in clinical practice, while technical and decision-making skills may deteriorate without repeated training opportunities.

Immersive virtual reality (VR) provides a potential educational solution. VR allows learners to practice high-pressure clinical scenarios in realistic, repeatable, and engaging environments, without the logistical burden of traditional simulation training. Recent studies demonstrate VR’s potential in health care education to enhance skill performance, knowledge acquisition, and clinical decision-making [14-16]. Using VR in medical training enables the user to be exposed to a highly realistic learning environment, as it combines the view of a real environment with additional virtual content through a screen or a VR headset [17]. Using VR as a teaching and training method can offer multiple advantages [16,18]:

- It allows learners to train independently at their own pace and location. No instructor is needed to observe the performance of trainees, and scenarios can be practiced as many times as desired.

- It helps train the essential individual components in management and provides interactive features such as voice, sound, and haptic feedback, as well as immediate learner feedback, which supports taking control of the learning process.
- It is cost-effective and affordable compared to other medical equipment. New training software programs can be updated remotely, and the hardware has a long operational expectancy.

We conducted a feasibility study in Tanzania (2024‐2025), across 3 hospitals with Public Health Laboratory-Ivo de Carneri as the facilitating local partner. The VR prototype was piloted on Meta Quest 3 headsets with 44 participants in clinical settings and showed that frontline HCWs, including doctors and nurses, who completed the pilot NEONATAL VR scenario in NR demonstrated improved clinical skills and high acceptability of the platform.

Despite growing evidence, gaps remain regarding VR’s effectiveness for NR training across different levels of HCWs, including medical doctors, and within European health care systems [17,19-22]. The NEONATAL randomized controlled superiority trial aims to investigate whether immersive VR simulation training, used as a supplement to traditional mannequin-based simulation training in Danish hospitals, improves NR performance. We hypothesize that repeated, deliberate practice under time pressure through immersive VR will enhance adherence to NR guidelines and improve HCWs’ NR performance, including rapid airway assessment, effective PPV, and timely initiation of critical actions.

## Methods

### Aim

The aim of NEONATAL is to study the effectiveness of VR-based simulation training in NR as a supplement to traditional simulation training for improving NR performance among doctors and nurses in Denmark. By the end of the study, we aim to have established a replicable framework that is scalable to other European countries.

The specific research objectives of NEONATAL are as follows:

- to investigate whether VR-based simulation training in NR improves clinical skills and serves as an effective supplement to traditional mannequin-based simulation training in 4 Danish hospitals (primary outcome).
- to investigate whether VR-based simulation training in NR on clinical knowledge is an efficient supplement to traditional mannequin-based simulation training (secondary outcome)
- to explore HCWs’ cognitive, emotional, and motivational responses during the VR-based simulation training (secondary outcome)
- to determine the feasibility and usability of VR-based simulation training in routine clinical training settings in Denmark (secondary outcome)

### Study Design

NEONATAL is a multicenter individually randomized, 2-arm, controlled superiority trial with parallel-group pretest-posttest design that was conducted from October 2025 to February 2026. Immersive VR-based simulation training as a supplement to traditional mannequin-based simulation training will be compared to exclusive traditional mannequin-based simulation training. We follow the SPIRIT (Standard Protocol Recommendations for Interventions Trials) guidelines (Checklist 1) [23].

### Study Setting, Population, and Recruitment

The study will be conducted at pediatric departments at 4 hospitals located in Region East, Denmark:

- Hillerød Hospital serves as a regional hospital in North Zealand. There are around 4000 births per year, and the hospital has a neonatal department with specialized level 2.
- Rigshospitalet serves as Denmark’s most highly specialized hospital and receives patients from Denmark, Greenland, and the Faroe Islands. There are around 6000 births per year, and the hospital has a neonatal intensive care unit with specialized level 4.
- Roskilde Hospital serves as a regional hospital in Region Zealand. There are around 3500 to 4000 births per year, and the hospital has a neonatal department with specialized level 2.
- Holbæk Hospital serves as a regional hospital in Region Zealand. There are around 1500 births per year, and the hospital has a neonatal department with specialized level 2.

Resident doctors and nurses from any of these hospitals with a clinical role involving neonatal care will be eligible for inclusion.

The inclusion criteria are as follows:

- Resident doctors with clinical function in neonatology
- Nurses with clinical function in neonatology

The exclusion criteria are as follows:

- No informed consent
- Change of workplace (to the nonstudy site) before endline or unable to participate in the study until endline
- Previously attended any formal training with VR
- Not fully proficient in Danish

Eligible candidates will be contacted by email as well as through informational meetings at the departments and invited to participate in the study. Informed and written consent, along with permission to record and store all video data and voice recordings for the research project, will be obtained from all participants upon enrollment.

Participant recruitment was completed in December 2025.

### Assessment Tools and Outcomes

A comprehensive evaluation strategy will assess clinical skills and clinical knowledge, as well as user experience, including cognitive, emotional, and motivational responses.

#### Primary Outcome

Clinical skills performance and adherence to the 2025 European Resuscitation Council Guidelines for Resuscitation (ERC)—Newborn Life Support (NLS) [24] will be evaluated during scenario execution at baseline and endline using mannequins. The evaluation will be performed by 2 assessors, using structured video observation checklists. The assessors are blinded for participant randomization. The NeoCheck, a validated 38-item checklist developed through a Delphi process, will be used to objectively assess participant performance during the NR simulation [25]. The maximum total score for NeoCheck is 48 points. Moreover, 4 additional global skills in logic and structured management, timing, practical applications, and errors will be rated, with a maximum total score of 8 points, as well as time to critical actions. Therefore, the total maximum score for the primary outcome is 56 points. The baseline and endline NeoCheck assessments are conducted as standardized individual simulation scenarios, in which each participant independently performs NR assessment and management tasks using the same assessment framework and scoring criteria to ensure comparability across assessments.

#### Secondary Outcomes

Clinical knowledge acquisition will be evaluated through preintervention and postintervention tests with multiple-choice questions (MCQs) aligned with the 2025 NLS curriculum [24]. The NEONATAL study group has developed the NR clinical knowledge test specifically for this project. The test consists of 23 MCQs, with 4 options and 1 correct answer.

Cognitive, emotional, and motivational responses will be evaluated at baseline and endline through the following:

- NASA Task Load Index (NASA-TLX) is a validated scale measuring perceived workload related to a specific task [26]. The workload score is based on 6 dimensions (mental, physical, and temporal demand, performance, effort, and frustration), and each dimension is scored from 0=very low to 100=very high, in increments of 5. This is based on the participants’ experience during the task. NASA-TLX has previously been forward-backward translated to Danish by 2 professional translators and tested in a Danish context [27].
- Intrinsic Motivation Inventory (IMI) is a validated questionnaire measuring intrinsic motivation related to a specific activity on a 7-point Likert scale ranging from 1=not at all true to 7=very true [28]. A 7-item version of the scale has recently been forward-backward translated to Danish and tested in a Danish context [27]. It includes the items from the subscale interest/enjoyment and will be used in this study.

The feasibility and usability of VR will be evaluated at endline through the following:

- System Usability Scale (SUS) is a validated questionnaire measuring the subjective usability of a product on a 5-point Likert-scale ranging from 1=strongly disagree to 5=strongly agree [29]. The SUS item scores are subsequently converted into a single SUS score on a scale from 0 to 100. SUS has previously been translated professionally to Danish and validated in a Danish context [30].
- Virtual Reality Sickness Questionnaire is a validated questionnaire measuring motion sickness in a VR environment on a Likert-type scale ranging from 0=none to 3=severe [31]. It includes 9 items covering symptoms of cybersickness.
- Records of training sessions and adherence, including the number and duration of VR sessions.

Further details regarding the study hypotheses, assessment tools, outcomes, and analysis methods are provided in Table 1.

**Table 1.** Assessment tools, outcomes, and analysis plan.

Outcome	Assessment tool	Participants	Time points	Hypothesis	Analysis method
Primary outcomes					
Clinical skills					
Clinical skills in NR^a^	NeoCheck [25] 38 clinical skills (preparation and resuscitation) 4 global skills Scoring: 0‐3 scale Total points: 56 Video-recorded, dual-rated, raters blinded	All (N=36)	Baseline (t_0_) Endline (t_3_)	Mean change in skills score will be significantly greater in intervention vs control group (superiority hypothesis)	Linear mixed effects model with hospital as a random effect and baseline score as covariate, intention-to-treat analysis
Time to critical actions	Video observation with time-stamping Time from birth to PPV^b^ initiation Time from birth to CPR^c^ initiation Time to scenario completion Right-censored at 900 (time to PPV and CPR) and 1200 seconds (time to complete scenario) if action not performed	All (N=36)	Baseline (t_0_) Endline (t_3_)	Intervention group will have higher hazard of performing critical actions (ie, faster performance) compared to the control group	Cox proportional hazards regression with robust SEs clustered by hospital, baseline time as covariate. Report HR^d^ with 95% CI and *P* value. Kaplan-Meier curves for visualization. Sensitivity analyses: AFT^e^ models with Weibull distribution, logistic regression for action completion (yes/no)
Secondary outcomes					
Clinical knowledge					
Clinical knowledge in NR	NR Knowledge Test 23 MCQs^f^ (4 options, 1 correct) Aligned with ERC^g^ NLS^h^ guidelines [24] Scored as percentage correct	All (N=36)	Baseline (t_0_) Endline (t_3_)	Mean change in knowledge score will be significantly greater in intervention vs control group	Linear mixed effects model with hospital as a random effect and baseline score as covariate
Cognitive, emotional, and motivational responses					
Perceived workload	NASA Task Load Index [26] 6 dimensions: mental, physical, temporal demand, performance, effort, and frustration Scoring: 0‐100 (increments of 5) Danish validated version	Intervention only (N=18)	Baseline postintervention assessment (t_1_) Endline (t_3_)	Exploratory: change in perceived workload from first to final VR^i^ exposure	Paired *t* test or Wilcoxon signed-rank test
Intrinsic motivation	Intrinsic Motivation Inventory [28] 7 items (interest/enjoyment subscale) Scoring: 7-point Likert scale (1=not at all true, 7=very true) Danish translated version	Intervention only (N=18)	Baseline postintervention assessment (t_1_) Endline (t_3_)	Exploratory: change in intrinsic motivation from first to final VR exposure	Paired *t* test or Wilcoxon signed-rank test
Feasibility and usability					
System usability	System Usability Scale [29,30] 10 items about perceived usability Scoring: 5-point Likert scale (1=strongly disagree, 5=strongly agree) Converted to 0‐100 score Danish validated version	Intervention only (N=18)	Endline (t_3_)	Exploratory: descriptive assessment of VR usability (benchmark: score >68=above average/acceptable)	Descriptive statistics (mean, SD, 95% CI)
Cybersickness	Virtual Reality Sickness Questionnaire [31] 9 symptoms of cybersickness Scoring: 0‐3 Likert scale (0=none, 3=severe) Scoring: 0‐100	Intervention only (N=18)	Endline (t_3_)	Exploratory: assess prevalence and severity of VR-related side effects	Descriptive statistics (frequency, mean, SD)
VR training adherence	VR session records Number of sessions completed Total training time	Intervention only (N=18)	Throughout intervention (t_2_)	Exploratory: describe VR training patterns and dose-response relationship	Descriptive statistics; correlation with skill/knowledge gains

a NR: neonatal resuscitation.

b PPV: positive pressure ventilation.

c CPR: cardiopulmonary resuscitation.

d HR: hazard ratio.

e AFT: accelerated failure time.

f MCQ: multiple-choice question.

g ERC: European Resuscitation Council.

h NLS: Newborn Life Support.

i VR: virtual reality.

### Intervention

The intervention consists of training in the NR VR training module. The VR training scenarios are standardized and identical for resident doctors and nurses. NR is inherently team-based, and although specific clinical responsibilities may differ between professions in practice, both nurses and physicians are expected to understand and follow the ERC NLS algorithm and perform core NR tasks during emergencies. Therefore, the VR scenarios focus on shared NR competencies relevant to both professional groups. The VR training module is developed in an iterative co-production process involving clinicians, educators, and Khora XR developers to ensure that the module meets real-world needs and is user-friendly in a medical environment. The iterative process ensures that the application and its content meet the expectations and requirements of HCWs. The NR scenario is an adapted version of the existing VR module content used in our feasibility study in Tanzania, to meet European health care standards.

The immersive VR scenarios use interactive features such as voice, sound (for instance, heart sounds), animated videos, and haptic feedback. During the VR scenarios, participants actively perform key steps in NR through hands-on interaction with the virtual environment and equipment. Learners assess the newborn’s clinical condition, select and prepare appropriate equipment, position the head, initiate PPV, reassess vital signs, and progress through the resuscitation algorithm based on the newborn’s clinical response. Interaction points are integrated into the virtual delivery room, enabling participants to initiate the assessment and treatment of the virtual patient through direct interaction with equipment and objects using handheld VR controllers. This allows participants to practice both procedural sequences and clinical decision-making under simulated time pressure. Information about the patient’s clinical condition, including vital signs, is provided during assessment. When interventions are made, the virtual patient’s clinical condition changes dynamically based on predefined changes in the patient’s vital signs and clinical state for each intervention. Furthermore, immediate visual and auditory feedback is integrated into the scenarios to reinforce correct actions and support reflective learning. Participants are able to change position in the virtual delivery room using teleportation. The VR scenarios force participants to reflect and train the essential individual components of NR, such as management of the airway and ventilation, and to train the sequence in the overall algorithms. The Meta Quest 3 headset is used to deliver the scenario.

The intervention in this study group is a low-volume high-frequency training in NR using VR. The VR training will take place on a weekly basis in short, under half-an-hour sessions over the intervention time period. The frequency of their individual VR training sessions, including duration, will be recorded during the intervention period. The low-volume high-frequency training model is used to allow HCWs to attend the training without compromising the patient service delivery by removing HCWs from their posts for training. The intervention group will be given a short tutorial and introduction to the VR simulation at baseline, but the VR scenarios are self-explanatory and will not be accompanied by further training during the intervention.

Throughout the intervention period, participants allocated to the intervention group will also participate in the department’s usual traditional mannequin-based simulation trainings. This guarantees that they receive the same routine NR training as the control group, with the addition of VR simulation training as a supplementary component. Traditional mannequin-based NR simulation training is not standardized by the study protocol and varies between participating departments with regard to frequency, participant composition, and simulation setting according to local clinical educational practice.

### Study Procedures

#### Data Collection and Integration of Training

Data collection will be conducted at all hospitals using the same methodologies and assessment tools. Data collection will be overseen by the principal investigator (PI) who will be supervised by senior researchers. All quantitative data will be entered into REDCap [32] by participants through tablets. The blinded assessors will be trained in the use of the NeoCheck checklist and the scoring system for the primary outcome. Videos will be stored on a secure storage. Primary outcome scores will first be recorded in password-secured Excel sheets, stored on a secure storage, and thereafter entered into REDCap.

No formal external monitoring of trial conduct is planned, as the study involves a low-risk VR-based educational intervention. Trial conduct, protocol adherence, and data completeness will be overseen continuously by the PI and the study team. Similarly, no Data Monitoring Committee has been established because the study has no anticipated serious adverse events or safety concerns requiring independent data monitoring.

Before the implementation of the study, interactive sensitization and awareness sessions about the utility of VR will be conducted with the site neonatologists, resident doctors, and neonatal nurses to support appropriate integration in routine clinical services. To promote participant retention and ensure complete follow-up, participants will be contacted by email to schedule end-line assessments at times that accommodate their work schedules.

#### Baseline Assessment (t_0_)

Baseline data will be collected for all participants, including gender, age, clinical experience, and prior experience with VR. Furthermore, an assessment of clinical skills and knowledge in NR will be performed at baseline. The participants will take part in simulated scenarios using mannequins to manage NR. The simulated clinical scenarios will follow the algorithm principles of the latest ERC guidelines for NLS [24]. The simulated scenarios will be recorded on video, and 2 blinded assessors will evaluate the performance, using a structured video observation checklist. Thereafter, participants will answer MCQs about their clinical knowledge on NR.

#### Baseline Intervention and Postintervention Assessment (t_1_)

Following the baseline assessment (t_0_), participants allocated to the control group will not participate in any further study-related activities. They will return for the end-line assessment after 6 to 8 weeks (t_3_). Participants allocated to the intervention group will, immediately after the baseline assessment (t_0_), receive a tutorial and introduction to the VR simulation training, followed by completing the VR scenarios in NR. They will then complete the NASA-TLX and IMI questionnaires to capture their cognitive, emotional, and motivational responses to the VR simulation training. This baseline intervention and postintervention assessment session is defined as t_1_.

#### Intervention Period (t_2_)

As described earlier in the *Intervention* section.

#### End-Line Assessment (t_3_)

At the 6 to 8 weeks follow-up (t_3_), all participants will answer a single-question questionnaire about how many traditional mannequin-based simulation trainings in NR they have participated in between baseline and endline, and participants allocated to the intervention group will also have their VR training time and frequency calculated. Furthermore, the intervention group will answer the NASA-TLX and IMI questionnaires, capturing their cognitive, emotional, and motivational responses to the VR simulation training, followed by the SUS questionnaire to assess the usability of VR, and the Virtual Reality Sickness Questionnaire to evaluate potential symptoms of cybersickness. Thereafter, clinical skills and knowledge in NR will be reassessed in all participants using procedures identical to those applied at baseline (t_0_). Finally, participants in the intervention group will be invited to participate in semistructured interviews to provide qualitative feedback on their VR training experience and the content of the VR modules. These interviews will be conducted concurrently with the randomized controlled trial. The qualitative component of this study will be reported in detail in a separate study protocol.

**Figure 1.**
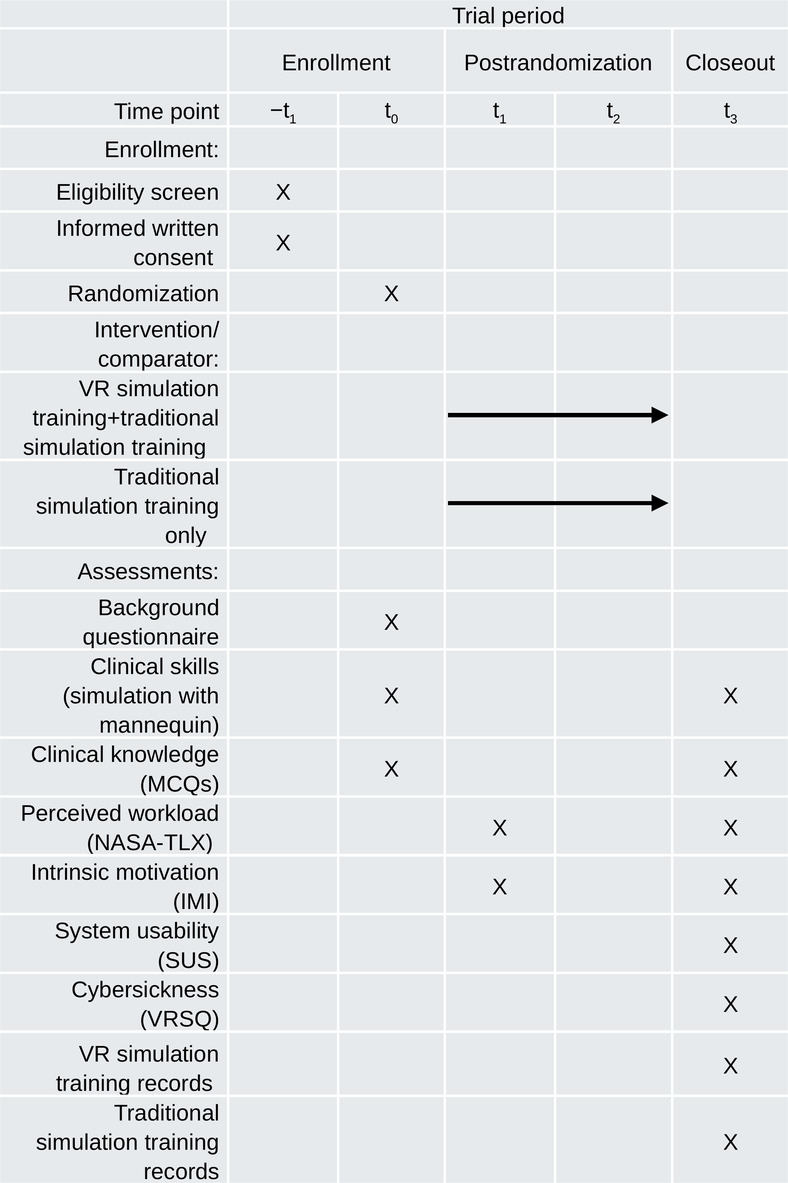
Participant timeline: schedule of enrollment, interventions, and assessments. IMI: Intrinsic Motivation Inventory; MCQs: multiple-choice questions; NASA-TLX: NASA Task Load Index; SUS: System Usability Scale; VR: virtual reality; VRSQ: Virtual Reality Sickness Questionnaire.

**Figure 2.**
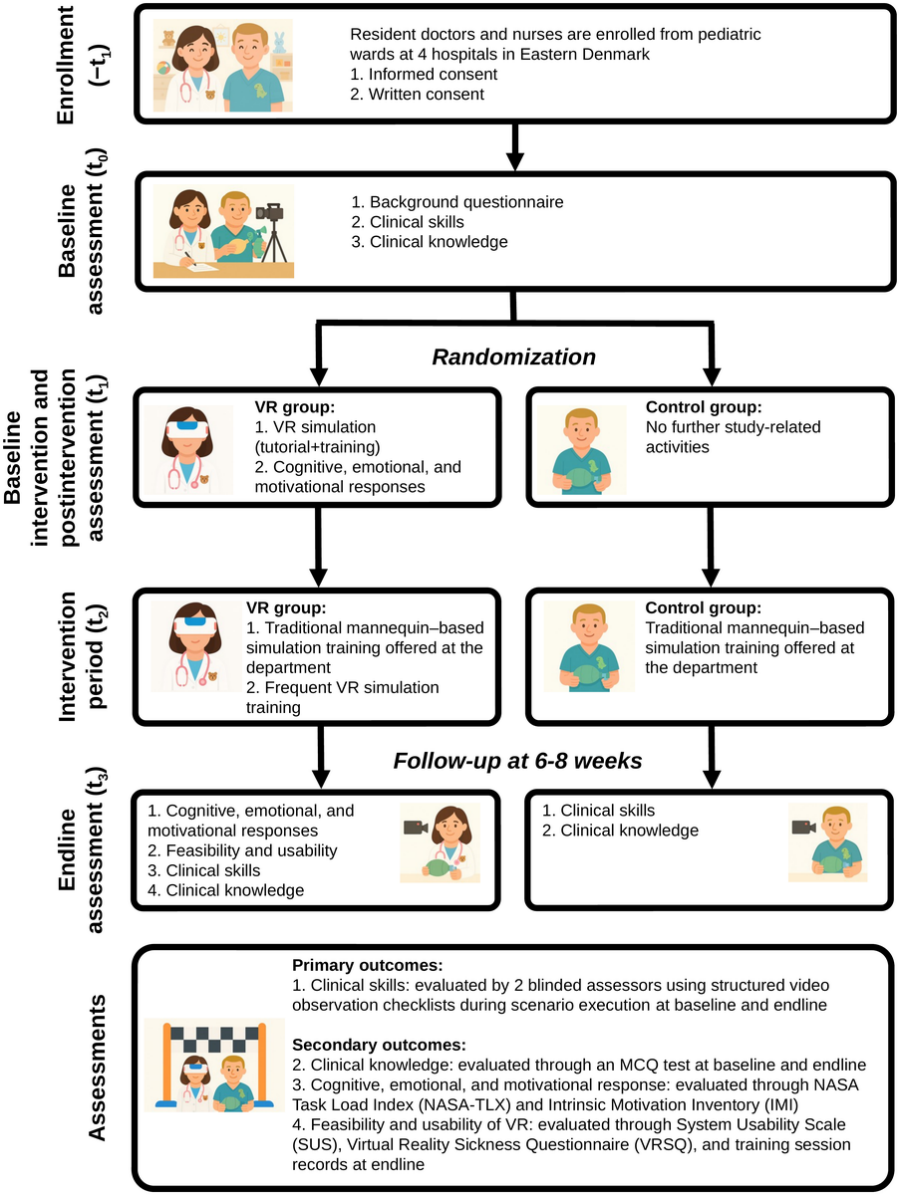
Study procedures. MCQ: multiple-choice question; VR: virtual reality.

The procedures including participant timeline for assessment and intervention phases are shown in Figures 1 and 2.

### Sample Size and Randomization

#### Sample Size

The primary outcome measure is based on a validated newly published 56-point assessment tool, the NeoCheck [25], for which no prior data exist regarding expected effect sizes or SDs. Sample size estimation was therefore based on data from our feasibility study assessing VR in a different clinical context (Tanzania), using a nonvalidated 25-point skills assessment tool. In that study, participants improved by 5.5 (22.1%) points of the maximum total score with an SD of 4.6 points (unpublished data). Although the assessment tool used in the feasibility study was not formally validated, its structure and scoring system are similar to the validated NeoCheck. To avoid underestimating variability, given some differences in scoring structure and validation status, the SD from the 30-point skills tool used in our feasibility study was conservatively scaled by the score range to the 56-point NeoCheck, yielding an SD of 10.3 points.

The NEONATAL study is designed as a 2-arm, parallel-group superiority randomized controlled trial with equal allocation (1:1). The target between-group difference was defined a priori as 20% of the maximum NeoCheck score (Δ=11.2 points). Sample size calculations were performed for a continuous primary outcome using a 2-sided significance level of 0.05% and 80% power. Under these assumptions (SD=10.3, Δ=11.2), 14 participants per group are required. To account for an anticipated 20% dropout or missing primary outcome data, the target sample size was increased to 18 participants per group, corresponding to a total sample size of 36 participants.

Furthermore, as the assumptions mentioned earlier, required for a conventional power calculation, are not fully reliable, we will use a pragmatic, time-limited inclusion strategy. All eligible resident doctors and neonatal nurses from the 4 participating pediatric departments will be invited to participate. During a predefined 8-week inclusion period, all staff who consent and are eligible to participate in the study will be enrolled, even if we reach above the number of 36 participants. We expect a high participation rate, and given the staffing levels at the participating departments, we anticipate reaching a sample size above the minimum of 36 participants.

#### Randomization and Blinding

Participants will, after the baseline assessment (t_0_), be randomized in a 1:1 ratio to one of 2 groups using restricted randomization with variable block sizes. Block randomization with randomly varying block sizes will be applied to reduce predictability. Randomization will be stratified by profession (2 levels) and recruiting hospital (4 levels). Although the anticipated sample size is less than 100 participants, the allocation list will be generated with a total length of 800 assignments to ensure coverage of all possible combinations of stratification factors. Details of block sizes, stratification structure, and the full allocation sequence will not be accessible to investigators involved in enrolling participants or assigning intervention.

The random allocation sequence will be generated by an independent data management provider (Frontal Lobe ApS) using the Sealed Envelope randomization service. Frontal Lobe ApS will not be involved in participant recruitment, enrollment, or assignment. The Sealed Envelope–generated allocation sequence will be uploaded into and implemented through the REDCap randomization module, which will be configured by Frontal Lobe ApS. Allocation concealment is ensured by the REDCap system, such that treatment assignments will be revealed only after a participant has been irreversibly enrolled and randomized. Investigators enrolling participants will not have access to the randomization sequence prior to assignment.

The 2 independent assessors of the videos of the primary outcome (clinical skills) will be blinded, and they will assess and rate the videos of the simulated scenarios without having any knowledge about participants’ allocation. The randomization is not blinded to the PI enrolling and assigning participants to the intervention, as well as implementing the intervention. However, the PI will not have access to the random allocation sequence.

### Data Analysis

All quantitative analyses will be conducted using R (R Foundation for Statistical Computing) software and will follow the intention-to-treat principle, with participants analyzed according to the randomized group. Missing data will be explored descriptively and handled using multiple imputation under a missing-at-random assumption, with complete-case analyses conducted as sensitivity analyses. All statistical tests will be 2-sided with *α*=0.05.

Descriptive statistics will summarize baseline characteristics by group, with continuous variables reported as median (IQR) and categorical variables as n/N (%). Prior VR experience and cybersickness scores will be described and explored as potential factors influencing participant engagement and study outcomes.

The primary outcome is NR clinical skills performance, assessed at baseline and endline using standardized checklist scores. All video-recorded scenarios will be independently rated by 2 blinded assessors; interrater reliability will be evaluated using the intraclass correlation coefficient (ICC), with ICC≥0.75 considered acceptable. If ICC <0.75, discrepancies will be resolved through consensus discussion or adjudication by a third rater. Final skill scores will be calculated as the mean of the 2 assessors’ scores unless adjudication is required. Between-group differences in end-line NR skill scores will be analyzed using linear mixed effects models with randomized group as a fixed effect, baseline score as a covariate, and hospital as a random intercept to account for clustering.

Time-to-critical NR actions (eg, time to initiation of PPV or cardiopulmonary resuscitation) will be analyzed using Cox proportional hazards models with hospital-level clustering, applying prespecified minimum clinically important time differences (eg, 30 s for PPV initiation) to support the interpretation of clinical relevance. Secondary continuous outcomes, including knowledge scores, will be analyzed using analogous mixed effects models. Outcomes collected only in the VR group (eg, cognitive workload, motivation, usability, and cybersickness) will be analyzed descriptively and, where measured longitudinally, using within-group comparisons. Given the exploratory nature of the secondary outcomes, we will not adjust for multiple testing, and the results will not be interpreted as confirmatory.

### Ethical Considerations

The study will adhere to the Declaration of Helsinki. Since this project requires HCWs and does not include direct interactions with neonates, ethical considerations primarily relate to the involvement of HCWs, the use of clinical settings, and the management of data collected during the study. The Danish Scientific Ethics Committee has granted an exemption to requiring ethical approval (F-25056089). The project has been reviewed and approved by PRIVACY, the Danish Data Protection Agency for the Capital Region of Denmark, which is responsible for GDPR (General Data Protection Regulation) compliance and legal oversight of personal data processing in research (p-2025‐19577). All activities will comply with Danish national laws and the ethical guidelines governing research in health care, including data protection laws.

Personal information from potential and enrolled participants will be collected and handled in accordance with applicable data protection regulations. Data will be collected and stored in a pseudonymized and secure manner. Only authorized study personnel will have access to identifiable information. During analysis, we will extract anonymized data from REDCap, which will be stored on a secure server. The video recordings will be transferred from local storage on the video camera to a secure storage. The videos will be deleted after the completion of data processing.

Participation will be voluntary, and only consenting resident doctors and neonatal nurses will participate in this project. Before any enrollment, the PI will inform the participants, by email as well as through informational meetings at the departments, about the purpose of the study, the nature of the VR training, and their rights, including the option to withdraw at any time. Written informed consent will be obtained from all participants upon enrollment. The consent form will be digital in REDCap and will outline the purpose of the study, how the data will be used, and measures to ensure confidentiality. Participant well-being is paramount, with support available should the simulation provoke emotional distress. Measures will be taken to ensure that participation does not interfere with patient care or disrupt clinical workflows.

The trial is registered on ISRCTN (identifier ISRCTN 43822066). Any protocol modifications will be documented and updated in the trial registry and communicated to relevant study personnel.

### Timeline

At the time of protocol submission, recruitment and baseline assessments have been completed. End-line assessments are scheduled for completion in February 2026. As our primary and secondary outcomes are entirely dependent on data collected at endline, the results cannot be generated until data collection is finalized. Data analysis is scheduled from March to July 2026, and the study results are expected to be published in autumn 2026. The details on the study timeline are provided in Table 2.

**Table 2.** Study timeline.

Phase	Timing	Status
Trial setup (registry, REDCap, randomization setup, and site preparation)	September to October 2025	Completed
Participant recruitment/enrollment	October to December 2025	Completed
Baseline assessments (t_0_)	October to December 2025	Completed
Start of intervention exposure for VR^a^ arm (t_1_)	October to December 2025	Completed
Intervention period (t_2_; VR low-volume high-frequency + usual simulation for all)	October 2025 to February 2026	Ongoing
End-line assessments (t_3_; 6‐8 wk postbaseline)	December 2025 to February 2026	Ongoing
Data extraction, verification, and cleaning	March to May 2026	Scheduled
Statistical analysis (primary+secondary outcomes)	March to July 2026	Scheduled
Reporting: results write-up and submission	July to September 2026	Scheduled

a VR: virtual reality.

## Results

The study was funded in July and October 2025, as well as in January 2026. Participant recruitment began in October 2025 and was completed in December 2025, with 66 participants enrolled. Data collection was completed in February 2026. Data cleaning and analysis will take place from March to July 2026, and the results are expected to be published in autumn 2026. This study will provide data on the effectiveness, feasibility, and usability of immersive VR simulation training as a supplement to traditional mannequin-based NR training in routine clinical education.

## Discussion

### Limitations of the Study Design

The NEONATAL study addresses an important gap in NR education by evaluating immersive VR simulation training as a supplement to traditional mannequin-based training. While the study is designed to assess effectiveness, feasibility, and usability, several methodological and practical considerations should be acknowledged.

First, the study was conducted within a limited geographic and organizational context, involving 4 hospitals in Region East, Denmark. Although these sites represent varying levels of neonatal care and clinical complexity, the findings may not be fully generalizable to other health care systems, particularly those with different organizational structures, staffing models, or resource constraints. However, the multicenter design and inclusion of both resident doctors and neonatal nurses enhance external validity.

Second, blinding is not feasible for participants or the PI delivering the intervention, which introduces a potential risk of performance and expectation bias. To mitigate this, the outcome assessment of clinical skills is based on video-recorded scenarios evaluated by blinded assessors using a validated checklist, thereby strengthening the internal validity of the primary outcome.

Third, the intervention focuses primarily on individual technical skills and algorithm adherence in NR. While VR enables immersive and repetitive practice of critical actions, it does not fully replicate team-based dynamics, leadership, or interprofessional communication, which are central to real-life resuscitation scenarios. As such, VR is positioned in this study as a supplement rather than a replacement for traditional team-based simulation training using mannequins.

Fourth, participants allocated to the intervention group receive supplementary VR-based training in addition to the department’s usual mannequin-based simulation training, whereas the control group receives usual training alone. Consequently, the study design does not fully distinguish the effects of the VR modality itself from the effects of increased training exposure. However, the study was intentionally designed as a pragmatic evaluation of VR as a scalable supplement to standard NR training, reflecting the intended real-world implementation of VR in clinical education.

Fifth, the follow-up period of 6 to 8 weeks limits the ability to assess long-term skill retention and transfer to clinical practice.

Sixth, although immersive VR training may offer advantages such as repeated self-directed practice, standardized scenario exposure, and flexible access to simulation training, the implementation of VR-based education still requires dedicated equipment, onboarding of users, and organizational support for integration into clinical training routines. However, the VR intervention in this study is designed as short, low-volume, high-frequency sessions using standalone VR headsets, which may reduce disruption to clinical workflows compared with more resource-intensive simulation approaches. Nevertheless, the feasibility and scalability of VR training may vary across health care settings and organizational contexts.

Finally, participation in the study is voluntary, which may introduce selection bias toward HCWs who are more motivated toward simulation-based learning or more comfortable with digital technologies and VR environments. Prior VR experience and cybersickness may also influence participant engagement and learning experiences.

### Integration, Scalability, and Long-Term Vision

A central ambition of NEONATAL is to ensure that immersive VR simulation training aligns with clinical needs and integrates seamlessly into existing health care workflows and training structures. The intervention is intentionally designed as a low-volume, high-frequency training model, allowing HCWs to engage in skills refreshment without disrupting patient care or requiring extensive instructor involvement.

While the initial testing phase is limited to Eastern Denmark, the VR modules are designed with scalability in mind. The content aligns with ERC guidelines and can be adapted to different languages, institutional protocols, and health care systems. User feedback, collected through quantitative measures and qualitative interviews, will directly inform the iterative refinement of the modules, ensuring continued clinical relevance and usability. The long-term vision of NEONATAL is to contribute to systemic and equitable improvements in neonatal emergency preparedness across Europe. By demonstrating effectiveness, feasibility, and usability in a real-world clinical setting, this study aims to position the NEONATAL VR platform as a robust, scalable, and market-ready solution for NR training. This approach supports rapid dissemination and implementation, particularly in settings where access to traditional simulation training is limited by time, cost, or staffing constraints.

### Dissemination Plans

The protocol, including the statistical analysis plan, is intended for publication in JMIR Research Protocols. The results of the study will be disseminated through multiple channels to maximize scientific, clinical, and societal impact. Primary findings will be published in peer-reviewed open access journals and presented at national and international conferences focusing on neonatology, medical education, simulation, and digital health. In addition, aggregated study findings will be shared with participating departments and hospital administrations to support local quality improvement initiatives.

Beyond academic dissemination, the project will engage with clinical educators, professional societies, and health care policymakers to promote the evidence-based integration of VR simulation into existing neonatal training frameworks. Insights from the feasibility and usability components will be used to inform implementation guidelines and best practices for VR-based training in routine clinical settings.

By combining evaluation with an implementation and dissemination strategy, the study aims to support the translation of immersive VR training into clinical practice.

### Patient and Public Involvement

Patients or members of the public will not be involved in the design, conduct, or reporting of this study, as the intervention targets health care professionals and focuses on clinical training.

## Supplementary material

10.2196/93439 Checklist 1 SPIRIT checklist.

## Abbreviations

ERC: European Resuscitation Council
GDPR: General Data Protection Regulation
HCW: health care worker
ICC: intraclass correlation coefficient
IMI: Intrinsic Motivation Inventory
MCQ: multiple-choice question
NASA-TLX: NASA Task Load Index
NLS: Newborn Life Support
NR: neonatal resuscitation
PI: principal investigator
PPV: positive pressure ventilation
SPIRIT: Standard Protocol Recommendations for Interventions Trials
SUS: System Usability Scale
VR: virtual reality
